# Lessons Learned from Immigrant Health Cohorts: A Review of the Evidence and Implications for Policy and Practice in Addressing Health Inequities among Asian Americans, Native Hawaiians, and Pacific Islanders

**DOI:** 10.1146/annurev-publhealth-060922-040413

**Published:** 2024-04-03

**Authors:** Alice Guan, AC S. Talingdan, Sora P. Tanjasiri, Alka M. Kanaya, Scarlett L. Gomez

**Affiliations:** 1 Department of Epidemiology & Biostatistics, University of California, San Francisco, California, USA; 2 Department of Health, Society, and Behavior, and Chao Family Comprehensive Cancer Center, University of California, Irvine, California, USA; 3 Department of Medicine, University of California, San Francisco, California, USA; 4 Helen Diller Family Comprehensive Cancer Center, University of California, San Francisco, California, USA

**Keywords:** Asian American, health equity, racial inequities, public health, cohorts, race and ethnicity

## Abstract

The health of Asian Americans, Native Hawaiians, and Pacific Islanders (AANHPI) is uniquely impacted by structural and social determinants of health (SSDH) shaped by immigration policies and colonization practices, patterns of settlement, and racism. These SSDH also create vast heterogeneity in disease risks across the AANHPI population, with some ethnic groups having high disease burden, often masked with aggregated data. Longitudinal cohort studies are an invaluable tool to identify risk factors of disease, and epidemiologic cohort studies among AANHPI populations have led to seminal discoveries of disease risk factors. This review summarizes the limited but growing literature, with a focus on SSDH factors, from seven longitudinal cohort studies with substantial AANHPI samples. We also discuss key information gaps and recommendations for the next generation of AANHPI cohorts, including oversampling AANHPI ethnic groups; measuring and innovating on measurements of SSDH; emphasizing the involvement of scholars from diverse disciplines; and, most critically, engaging community members to ensure relevancy for public health, policy, and clinical impact.

## INTRODUCTION

**Figure 1:**
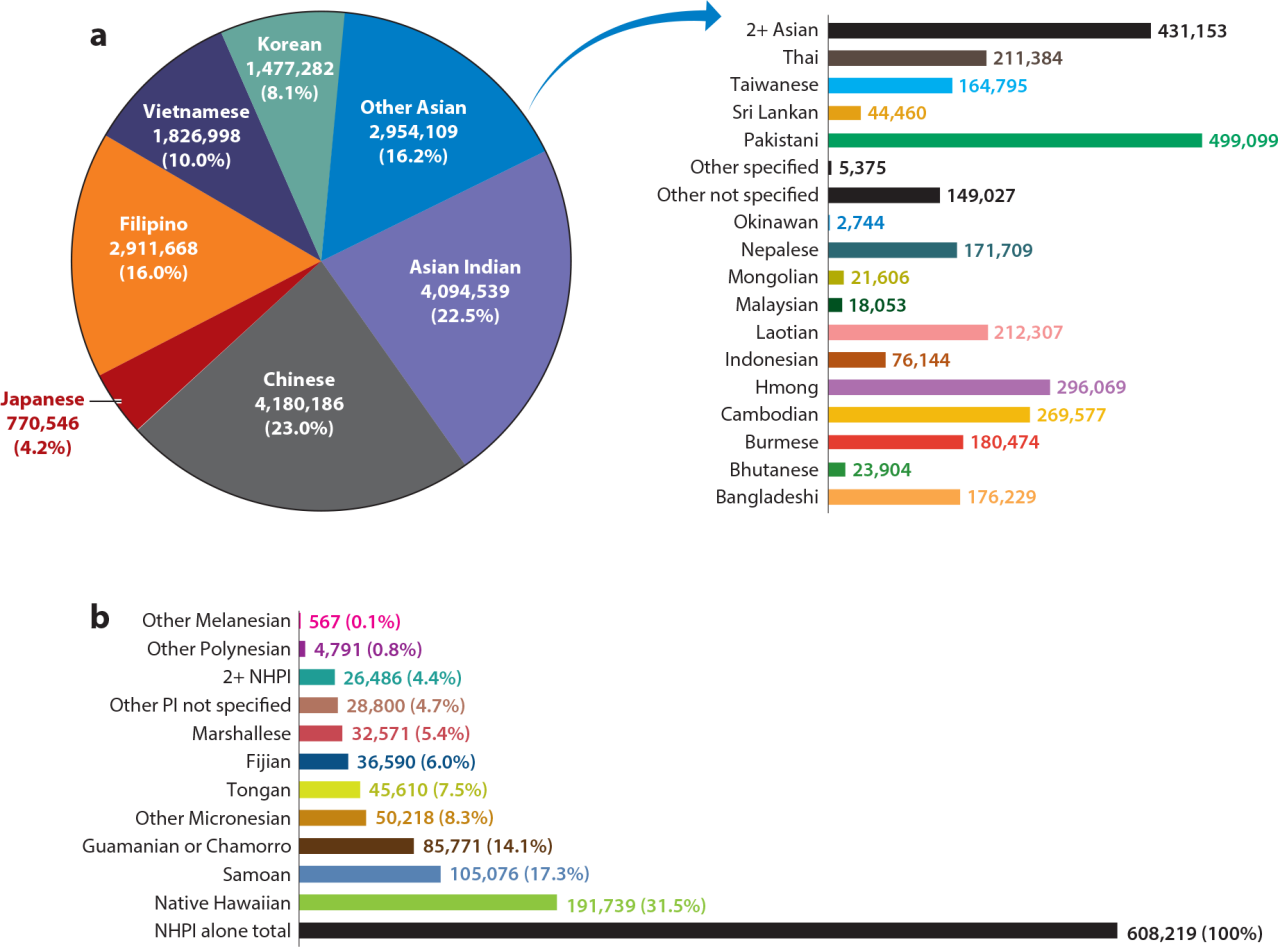
(*a*) Distribution of Asian American ethnic groups (alone) in the United States, 2017. (*b*) Distribution of Native Hawaiian and Pacific Islander (NHPI) ethnic groups (alone) in the United States, 2017. Figure panels created using data from AAPI Data, based on 2017 American Community Survey one-year data (for Asian Americans, https://aapidata.com/stats/national/national-detailed-origin-aa-aj/; for Native Hawaiian/Pacific Islanders, https://aapidata.com/stats/national/national-detailed-origin-nhpi-aj/).

**Figure 2:**
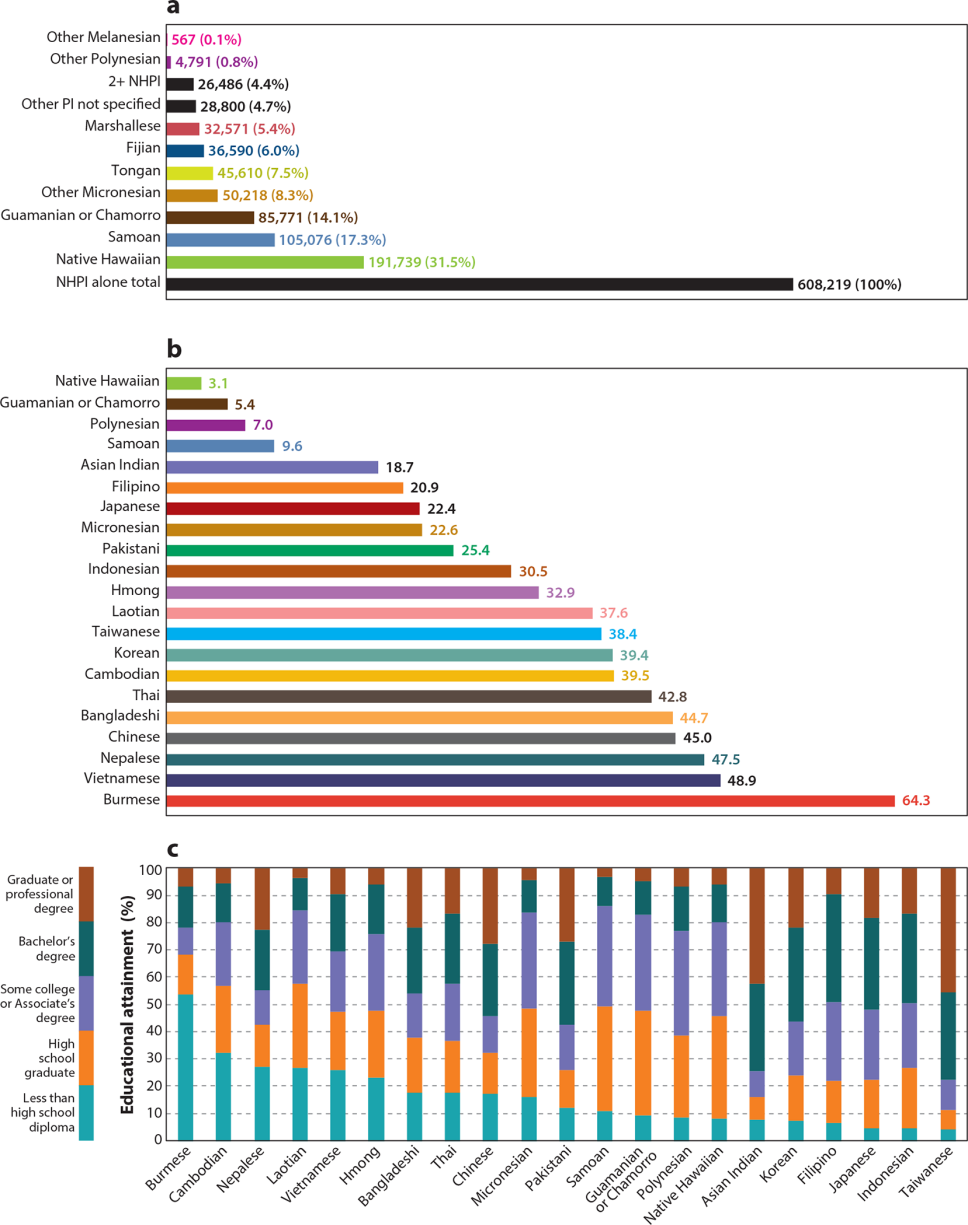
Prevalence of selected sociodemographic characteristics by Asian American, Native Hawaiian, and Pacific Islander (AANHPI) ethnic group. (*a*) Percentage of population who speak English less than very well, (*b*) percentage living in poverty, (*c*) highest educational attainment. Figure panels created using data from AAPI Data, based on 2017 American Community Survey (https://aapidata.com/stats/national/).

Asian Americans, Native Hawaiians, and Pacific Islanders (AANHPIs), numbering more than 25.5 million in the 2020 US Census, are two of the fastest growing racialized minority groups in the United States, with the population of Asian Americans (AAs) alone and in combination (mixed-race Asian individuals) increasing by 55.5% between 2010 and 2020 and the population of Native Hawaiians and Pacific Islanders (NHPIs) alone and in combination increasing by 30.8% (51). Up until the 2010 Census, the AANHPI population was often combined into one group, and, despite disaggregating AAs separately from NHPIs in the 2010 Census, many statistics are still reported with aggregation. In fact, AANHPIs derive from more than 40 countries of origin and represent more than 100 different languages and dialects (Figure 1). As a result of their diverse histories of immigration to, or colonization by, the United States, the AANHPI population is incredibly heterogeneous in all possible ways—culture, lifestyles, socioeconomic status, etc. (Figure 2). For example, the AANHPI population encompasses both the highest and lowest ethnic group–specific household income levels ($123,700 among Asian Indian and $49,854 among Bhutanese) of any race or ethnic group in the United States (13, 14). More than 75% of Asian Indians are college graduates, compared with ~15% of Laotians, Samoans, and Micronesians. Nearly one-fourth of Burmese are living in poverty, in contrast with 8% of Japanese (14).

Moreover, historical residential exclusionary laws and practices, and patterns of settlement into ethnic enclaves, created variable exposures to structural and social determinants of health (SSDH) (77), defined as the interconnected and mutually reinforcing social, economic, and public policies and programs as well as the conditions (social and built environments) in which people live (116). AANHPIs have long experienced discrimination, manifesting as structural, institutional (e.g., housing, health care), and interpersonal biases due to their marginalized statuses at the intersections of ethnicity, language, immigration status, religion, and culture (1, 25, 33, 50, 67–69, 127). Structural discrimination produces stark patterns of residential segregation and inequities in neighborhood exposures, including housing, unemployment, poverty, crime, environmental pollutants, access to health care, and social isolation (1, 33, 50, 67–69, 127). Yet ethnic enclaves may provide to its residents ties to places of worship, cultural affinity networks, and community centers (58), providing buffers to structural and social stressors (81) and facilitating access to information on health care services and culturally and linguistically appropriate resources (2).

Most AAs (59%) are immigrants; some came to the United States as refugees, experiencing both pre- and postmigration trauma (34–36, 113). AAs have experienced high levels of discrimination (35, 41, 89), amplified by the COVID-19 pandemic (24, 47, 48, 119), with negative impacts on health behaviors and health care access (82, 107). In contrast, NHPIs are largely indigenous or freely entering populations (e.g., due to the Compacts of Free Association with nations in Micronesia) in the United States, but the enduring impacts of colonization as well as military occupations throughout Polynesia, Micronesia, and Melanesia can be seen in the lower economic status and educational attainment of NHPIs in the United States. The lack of disaggregated NHPI data continues to be a particularly insidious form of structural racism (85). For example, more than one-third of states reporting COVID-19 cases and deaths in 2020 failed to report disaggregated NHPI data; it was only with community advocates and leaders initiating their own tracking that the nearly three times higher likelihood of death among NHPIs, as compared with Whites, was described in the literature (83, 100).

These structural, historical, and sociodemographic differences translate into observed heterogeneity in disease risks. In 2021, scientists convened for a National Institutes of Health (NIH) workshop, cochaired by author Alka Kanaya, to identify knowledge gaps and discuss research opportunities for AANHPI health across multiple exposure and disease domains. The workshop report summarized the existing evidence on the epidemiology of several health conditions across AANHPI populations (56). Although a pervasive theme was the sparse data among disaggregated AANHPI subgroups, there were some clear signals showing gradients in disease risk and health disparities across AANHPI groups. Several metabolic conditions (e.g., obesity, type 2 diabetes, nonalcoholic fatty liver), cardiovascular diseases, and cancer types have higher incidence and prevalence in specific AANHPI groups versus other race/ethnic groups, with significant differences compared with the majority non-Hispanic White population in the United States (56).

An example that demonstrates differences in health risk as well as divergence in disease outcomes among AANHPI ethnic groups is the case of type 2 diabetes. Type 2 diabetes is highly prevalent among NHPIs, followed by South Asians and Filipinos, and several other Southeast and East Asian groups, with most AANHPI groups having higher prevalence than non-Hispanic White adults (19, 40, 59). However, metabolic disorders that often lead to type 2 diabetes (such as obesity and fatty liver) differ among the three groups with the highest diabetes risk; NHPIs have more obesity but lower fatty liver prevalence than what has been reported among South Asian and Filipino populations (6, 80, 110, 112). Moreover, these three groups also diverge in cardiovascular outcomes of diabetes, with higher heart disease among NHPIs and South Asians and higher stroke risk among Filipinos (19, 112). A lack of longitudinal studies that include substantial numbers of NHPIs, South Asians, and Filipinos limits public health researchers’, policy makers’, and community organizations’ understanding of the social, cultural, behavioral, and biologic factors that explain these disparate disease risk factors and outcomes.

For most AANHPI groups, cancer has been the leading cause of death for decades (44). Certain ethnicities also have unique and dynamic patterns in cancer risk (39, 120). For example, among San Francisco Bay Area AAs, breast cancer risk was recently observed to be higher among immigrants relative to U.S.-born individuals, with an even more pronounced pattern among more recent birth cohorts and among younger women, signaling a potential shift in breast cancer risk (49, 84). AA women have almost twofold higher incidence of lung cancer in never smokers relative to non-Hispanic White women, with high proportions of never smokers among lung cancer patients, up to 80% among Chinese women (26, 28). The Korean population is five times more likely to develop gastric cancer than non-Hispanic Whites, a result of higher *Helicobacter pylori* infection rates and unique dietary factors (74). Liver cancer rates among Southeast Asians and cervical cancer among Vietnamese women are among the highest in the world (39), in part due to the high prevalence of hepatitis B virus and human papillomavirus infections (123), respectively.

These variable and dynamic disease rates across AANHPI populations alongside their unique risk factor distributions offer the potential for novel scientific insights. Among observational epidemiologic study designs, the prospective cohort is the most rigorous and minimizes biases inherent to study designs such as cross-sectional or case-control studies. Longitudinal cohorts where exposure is assessed, ideally with multiple assessments over time, prior to the occurrence of incident disease are less susceptible to recall bias and are more robust for inferring causal exposure–disease relationship. In this article, we have reviewed prospective, longitudinal cohorts of AANHPI populations, selected seminal findings and lessons learned from them, and provided suggestions for future AANHPI cohorts for informing policies and practices to address health inequities. We focus specifically on the findings related to SSDH in these cohorts, given that they can serve as leverage points for intervention or policy change.

## METHODS

### Description of Cohorts Included in This Study

This review aims to describe longitudinal, observational cohorts in the United States that purposively sampled specific AANHPI groups, with attention to the inclusion of immigrant populations and to the capture of immigration and acculturation characteristics, in addition to other phenotypic data collected from participants over time. We did not exclude any cohorts on the basis of exposure or disease/outcomes data collected. Due to the limited number of cohorts, we did not conduct a formal search of existing databases; instead, expert knowledge from the coauthor team was used to identify cohorts that met these criteria. Cross-sectional studies, such as the National Latino and Asian American Study and other health system cohorts (e.g., Kaiser Permanente, Sutter Health) that have been linked to registries, claims, and/or vital statistics records allowing for prospectively assessed outcome ascertainment and longitudinal analyses, were excluded because they did not collect primary or repeated exposure measurements from participants over time. Finally, we excluded non-English language cohorts and US-funded studies that recruited participants in other countries (e.g., the Shanghai Chinese Health Study). We ultimately included a total of seven cohorts: the Children of Immigrants Longitudinal Study (CIL), the Honolulu Heart Program (HHP), the San Diego Filipino Health Study (FHS), the Japanese American Community Diabetes Study ( JACD), the Multiethnic Study of Atherosclerosis (MESA), the Multiethnic Cohort (MEC), and the Mediators of Atherosclerosis in South Asians Living in America (MASALA).

### Cohort Characteristics

For each cohort included in the review, we utilized various sources such as cohort websites, study design manuscripts, protocol papers, and communications with principal investigators to extract data in three main areas. First, we extracted data on cohort characteristics, including the years of data collection, the geographic regions covered, the number of participants who were recruited at baseline, and the specific AANHPI ethnic groups included. Next, we extracted information on SSDH that is relevant to the AANHPI communities, including socioeconomic status (SES), measures of acculturation, cultural or social constructs specific to AANHPI populations, self-reported stressors, health care, and neighborhood factors. Finally, we quantified the contributions of each cohort by describing the number of publications that utilized the cohort data and the primary health outcomes that were evaluated, using our collective expertise in measurement to selectively highlight key gaps in evidence.

## RESULTS

### Cohort Characteristics

Table 1 describes the key findings from the seven longitudinal cohort studies. Most cohorts (71%) were incepted prior to 2000, with the exception of MESA (2000–2002 baseline exam) and MASALA (2010–2013 baseline exam). The three states with the most representation across cohorts were California, Hawaii, and Washington. Only three cohorts included participants outside of these states: CIL, which included participants in Florida (though that sample captured primarily the Hispanic population, which was another target for the cohort); MASALA; and MESA. The average number of baseline participants was 2,211 and ranged from 453 (FHS) to 215,251 (MEC). Most cohorts (86%) were composed predominantly of a single AANHPI ethnic group.

### Measures of Relevant Structural and Social Determinants of Health

#### Socioeconomic status.

The most common measures of SES included educational attainment, employment status, occupation, and family income. Three studies also asked about home ownership.

#### Acculturation.

The most common measures of acculturation were nativity and years lived in the United States. Language questions were also common and included assessments of English proficiency and languages spoken at home (CIL, HHP, MESA, MASALA). Notably, due to colonialism for both the Filipino and South Asian populations, English language proficiency was not a relevant indicator of acculturation. As such, the FHS cohort asked participants their preferred language for consuming TV, radio, and print media, and MASALA asked several other questions to measure cultural beliefs and practices (87, 90). Four studies also included questions related to cultural diets and foods (e.g., the Japanese Diet Score included in the HHP, which provided a frequency of Japanese foods consumed relative to all foods consumed).

Two studies (CIL and HHP) included more extensive questions to assess less commonly used dimensions of acculturation. These included the importance of having a tight-knit cultural community (e.g., preferring employment, friendship, and engagement with people from one’s own country), opposition to Whiteness (e.g., opposition to marriage with White Americans), and connection to one’s own national background (e.g., pride in one’s native country). Notably, only the CIL evaluated these constructs longitudinally, while the HHP included these assessments in only one wave of data collection.

#### Cultural and social constructs specific to AANHPI populations.

Four studies included some measurement of cultural and social constructs specific to AANHPI populations (CIL, HHP, FHS, MASALA). Constructs measured included the importance of maintaining traditional cultural values, attending social or cultural gatherings, religious or spiritual beliefs, and valuing proximity to family when making decisions about residence or work. Notably, with the exception of CIL, cohorts that focused recruitment on specific AANHPI ethnic groups provided more tailored assessment of these questions. For instance, in the HHP, questions about cultural and social constructs were specifically named and identified Japanese holidays and religious practices.

#### Self-reported social stressors.

A majority of the cohorts included a measure of discrimination, with most asking follow-up questions to determine whether participants perceived the discriminatory experience as being due to their race or ethnicity.

#### Health care.

Six of the cohorts included measures of health insurance or access to care.

#### Neighborhood environments.

Many of the cohorts measured participants’ self-reported perceptions of neighborhood safety or racial composition. In addition, many of the studies included data on participant residential addresses and changes in residential addresses over time, and investigators used these to link to geospatially referenced data on structural, social, built, and physical neighborhood environments.

#### Cohort contributions.

The average number of publications produced using cohort data was 570 and ranged from 27 (FHS) to 2,154 (MESA). A majority (86%) of cohorts were formed with the primary objective of evaluating specific health outcomes: These included coronary heart disease, stroke, cancer, diabetes, and other chronic diseases. Only CIL evaluated adaptive outcomes, as the purpose of the study was to quantify experiences of immigration. See Table 2.

### Key Findings on Structural and Social Determinants of Health Across Studies

#### Socioeconomic status.

Low SES was consistently associated with poor health outcomes across different studies and populations, whether operationalized to capture individual-level and/or neighborhood-level SES. For instance, studies emerging from the FHS and JACD found evidence that lower educational attainment was associated with increased risk for diabetes (4, 76). Data from MESA and MEC also provide evidence that indicators of low SES were associated with worsened cognitive performance, biological indicators of inflammation and stress, and mortality (31, 45, 92, 105, 109, 114). Education and family income were high among the majority Asian Indian population in MASALA, and, contrary to other cohorts, South Asians showed fewer associations between these socioeconomic measures and health status.

#### Acculturation.

Evidence on the effects of acculturation on health were also largely consistent across studies and populations. In general, findings from HHP and MESA suggest that higher levels of acculturation, measured by years in the United States, were harmful to health (22, 30, 43, 78, 97, 99, 128, 129). However, one study from MESA found that for Chinese individuals, acculturation could have been potentially protective (29). Evidence from CIL also suggests that factors such as parental involvement could ameliorate intergenerational tensions caused by a lack of acculturative concordance between parents and their children (130, 131). Furthermore, data emerging from studies that focused on specific AANHPI ethnic groups allowed for additional nuance and the ability to build on concepts of acculturation over time. For instance, investigators characterized three distinct profiles of acculturation among South Asian communities in MASALA and subsequently described associations among these profiles with regard to health outcomes (e.g., cardiometabolic profiles, symptoms of depression) (90, 91). In contrast with other cohorts reviewed here, the MASALA study found that women in the integration and assimilation acculturation classes (with moderate or higher acculturation to the United States) had fewer cardiometabolic risk factors (3).

#### Cultural and social constructs specific to Asian American populations.

Evidence from these cohorts on the influence of traditional, cultural, or social factors on the health of AANHPI populations was limited. Traditional family values were one area that investigators explored across studies. For instance, findings from CIL suggest that traditional beliefs about family values differed between male and female AAs (121). In addition, data from the HHP suggest that traditional beliefs about family obligations and preferences for elder care can help to explain why Japanese Americans have greater rates of hospital deaths (versus nursing home deaths) (9).

Another area of culture explored in the MASALA study related to religiosity and spirituality, with regard to the role that these factors play on health. For instance, high religiosity and spirituality were found to be positively associated with self-rated health, but religious affiliation was also found to increase the odds of being overweight/obese among Hindus, Sikhs, and Muslims and of higher cholesterol levels among Muslims (10, 46). Religious struggles were also found to influence the relationship between protein biomarkers and the risk of cardiovascular disease (93).

#### Self-reported social stressors.

Research from MESA and MASALA provides evidence of the harmful effects of discrimination (e.g., associations with increased tobacco and alcohol use, all-cause and cardiovascular mortality) (12, 62, 73, 88, 126), but it also showed that the effects of everyday experiences of discrimination do not explain other hypothesized outcomes, such as diabetes and consumption of fruits/vegetables (61, 126). Furthermore, findings from CIL suggest that perceptions and experiences of discrimination, specifically for youth, may differ based on biological sex, an area that needs further exploration (79, 124).

#### Health care.

We found limited exploration of health care in published studies across cohorts. However, existing evidence from MESA suggests that health insurance of any type was associated with lower fasting glucose levels among individuals with diabetes (38), though the Chinese participants in this cohort were six times more likely to have no health insurance coverage compared with White participants (31).

#### Neighborhood environments.

Evidence on the impact of neighborhood environments, which can include attributes of structural, social, built, and physical environmental factors, on health outcomes across MESA, MEC, and MASALA were largely consistent. For instance, MESA and MASALA studies found that better physical environments such as those with walk spaces were associated with lower body mass index, higher probability of consuming organic foods, and increased walking activity (61, 86). In addition, studies from MESA and MEC both found that air pollution was associated with negative health outcomes (e.g., acceleration of atherosclerosis, lung cancer risk) (18, 42). However, studies on neighborhood ethnic concentrations were more mixed, suggesting that ethnic enclaves may have both promoting and deleterious health effects. While evidence from MESA and MEC suggests that residence in segregated or immigrant communities was associated with worse health outcomes (e.g., breast cancer risk) (63, 98), findings from CIL suggest that coethnic communities were an important resource specifically for monolingual children (75).

## DISCUSSION

This review highlights seminal features and selected findings on the sociocultural, interpersonal, and neighborhood-level SSDH measures with selected health outcomes from seven large longitudinal epidemiologic cohorts that included substantial numbers of AANHPI populations. These cohorts have shown important differences in demographic, social, cultural, and environmental dimensions from other US racial and ethnic groups, and across and within AANHPI ethnic groups, and how these differences are related to several health outcomes. Several themes show associations between SSDH and health outcomes similar to what has been observed in other non-AANHPI populations. However, there are several notable areas of divergence among specific AANHPI groups, with different patterns of association between acculturation and other social factors and health outcomes. These findings exemplify the vast diversity among the AANHPI subgroups and how aggregating all groups together obscures important differences by failing to expose areas of health disparity and perpetuating inequities in health.

Three of these longitudinal cohorts (HHP, MESA, and MASALA) were funded by contracts or grants from the National Heart Lung and Blood Institute. These three cohorts have followed Japanese American, Chinese American, and South Asian American individuals over several years with goals for determining etiologic risk factors for the occurrence of cardiovascular disease outcomes. These cohorts included predominantly a single ethnic group (HHP with Japanese American men, MESA with primarily Chinese Americans, and MASALA with primarily Asian Indians). Since MASALA was designed to be comparable to MESA with regard to methods and measures, several papers have directly compared these two AA samples, highlighting risk differences in behaviors, body composition, cardiovascular risk factor profiles, subclinical atherosclerosis burden, disease incidence, and modifiable risk factors for each group (e.g., physical activity, dietary patterns, and other neighborhood factors). Having cohorts in which multiple different AANHPI groups are included in substantial numbers and are followed over time allows direct comparison of risk profiles and outcomes that are otherwise challenging to compare across studies using different sampling strategies, eligibility criteria, and measures collected in each study. In contrast, a strength of the single ethnic group cohort studies is that many investigators have added more specific sociocultural and contextual measures tailored to the cultural group under study, thereby providing richer data that can be used for defining levers for intervention and implementation within each group.

As the only cancer epidemiologic cohort with purposive sampling of AANHPI populations, the National Cancer Institute (NCI)-funded MEC includes nearly 57,000 Japanese and 14,000 Native Hawaiians. There are several thousand individuals of other AANHPI ethnic groups as well, though not purposively (122). The MEC has made seminal contributions to the understanding of cancer etiology and, increasingly, of other diseases for these two AANHPI groups; moreover, given its exceptionally large sample size of each ethnic group, MEC is well powered for subgroups analyses, such as disease risks by ethnicity, sex, age, and other characteristics. A notable feature of the MEC is the recent effort to expand the data infrastructure to include neighborhood contextual data through geocoding residential addresses over time, facilitating multilevel studies of the impacts of environmental factors on disease incidence, and evaluating interactions with genetic factors (18, 20, 21, 27, 109, 115). However, as three other racial and ethnic groups were purposively sampled in the MEC, surveys items are not specific to particular groups, and, thus, culturally and ethnically specific constructs are not included.

Several of these cohort studies have outlined key demographic and sociocultural differences and highlighted several areas with significant health disparities that deserve more detailed focus. Several gaps in the literature were identified at the recent NIH workshop, and specific strategies that can help to fill these gaps were discussed (56). In the domain of sociocultural factors, some fruitful areas of research include studying the effects of immigration and acculturation across immigration waves and generational status to determine earlier nodes of intervention. None of the existing cohorts have measured the context of or reasons for immigration (e.g., immigration due to political conflict, employment) or political relationships between the United States and their countries of origin and the impact of stress related to immigration or acculturation. Discrimination has been studied with tools that were neither created nor validated among AANHPI populations and have not included measures of discrimination based on language, country of origin, culture, caste, color, religion, or ethnic dress. Traditionally less studied but perhaps the most important as a fundamental cause of health inequities (8, 125), structural and institutional racism among AANHPI populations has recently received more attention, including from the US White House, as a result of anti-Asian hate rhetoric and crimes. However, structural racism against AANHPIs has had a long and insidious history in the United States (35). Novel approaches using, for example, mortgage data and Twitter data can capture structural racism against AANHPIs in different domains (32, 48, 94).

Interpersonal and structural factors that influence health behaviors such as familial structures, social networks, community and civic involvement, and health care access and utilization have important influences on health behaviors, but these domains are understudied. Individual-level behaviors such as diet, substance use (tobacco and other cultural tobacco products, alcohol, recreational drugs), physical activity, and sleep have also not been measured well in these diverse communities. Several of these behaviors can be objectively measured with novel biomarkers and digital health tools (e.g., metabolic biomarkers of diet and substance use, activity and sleep trackers) in addition to culturally tailored survey items. In addition, longitudinal assessment of health care access and health outcomes, particularly given evolving health care policies and impacts on access (e.g., Affordable Care Act), which is critical for evaluating policy change and increasing care, is an understudied area.

These seven cohorts demonstrate the value of longitudinal observational cohort resources for advancing scientific knowledge about disease risk factors among the growing AANHPI populations, generating practice- and policy-relevant research for addressing disease burden in specific ethnic groups, and providing insights into the discovery of disease risk factors. However, the fact that there were only seven such cohorts that we could include even with our broad criteria is, in itself, a glaring health inequity. New cohorts of AANHPI populations are needed to address the continuing disease burdens and disparities unequally and inequitably borne by distinct AANHPI groups. Next-generation cohorts should learn from the successful and fruitful examples but also build on the earlier cohorts, including purposive oversampling of AANHPI ethnic groups; assessment of SSDH, including the domains described in this review; and assessment of relevant life experiences, including the immigrant experience and immigration factors. These new cohorts should engage scholars from different disciplines to bring relevant perspectives from fields such as history and cultural anthropology, sociology, demography, psychology, political sciences, genetics, clinical medicine, etc. The most important perspective, however, that absolutely needs to be engaged in any new AANHPI cohort is that of the community. The value of community-engaged research has been well documented and cannot be understated; however, in the context of an AANHPI cohort, community engagement is essential for ensuring that the important and relevant questions are asked and asked in the right way and that results are disseminated for maximal public health, policy, and clinical impact.

We are heartened that the NIH recently provided directed funding opportunities with a Notice of Special Interest (NOT-HL-23-001) for epidemiologic studies among AANHPIs and two Requests for Application (RFA-HL-23-015 and RFA-HL-23-016) to establish a new, community-based, multiethnic, longitudinal cohort. This cohort would include at least 4-5 different AANHPI national origin groups in large numbers to enable researchers to fill important gaps and test scientific hypotheses that were identified at the NIH workshop. The ultimate success of these new endeavors will be measured by authentic and meaningful community engagement and long-term improvements in the health status of AANHPI populations.

## Figures and Tables

**Table 1:** Cohort characteristics and measures of structural and social determinants of health collected by each AANHPI cohort

Cohort	Number of AANHPI participants at baseline (*N* total, if other groups included)	Socioeconomic status	Acculturation	Self-reported social stressors	Health care	Neighborhood environment	Number of publications^a^	Primary outcomes of interest
CIL; 1992–2003; Miami/Ft. Lauderdale, FL, San Diego, CA (102, 103)	1,771 Vietnamese, Laotian, Cambodian, Chinese, Hmong, Other Asian (5,262 total)	Amount saved for child’s education Educational attainment Employment status and occupation Home ownership Self-reported economic situation Income Use of government assistance	Valuing family proximity Nativity Years in the United States English language proficiency Language spoken at home Plans to stay in the United States/satisfaction with US life Importance of having a tight-knit cultural community Relation to the “American way of life” Connection to national background Opposition to whiteness	Discrimination Perceptions of racial inequality Governmental contact (e.g., use of government aid)	Health insurance	Neighborhood safety Neighborhood racial composition School racial composition	318	Adaptation outcomes (e.g., educational attainment, political attitudes and participation, delinquency and incarceration, plans for the future)
HHP; 1965–2015; O‘ahu, HI (52, 71, 95, 106)	8,006 Japanese American men	Educational attainment Employment status and occupation Home ownership	Valuing family proximity Engagement with cultural practices Engagement with religious practices Cultural mores Nativity Years in the United States English language proficiency Native language Japanese Diet Score (ratio of sum frequency of Japanese food consumption to all foods consumed) Importance of having a tight-knit cultural community Feelings about different generations of immigrants Seeking culturally tailored services Opposition to whiteness	Discrimination	ND	Neighborhood racial composition	284	Coronary heart disease and stroke
UCSD FHS; 1994–2007; San Diego, CA (5, 7)	453 Filipina women	Educational attainment Employment status and occupation Poverty	Importance of keeping traditional practices Nativity Years lived in the United States Race/ethnicity of friends Language of TV, radio, and print media	Discrimination Self-reported stress Social connectedness	Access to care	ND	27	Diabetes and cardiovascular disease
Seattle JACD; 1983–1985 at baseline; Seattle, WA	Japanese Americans	ND	Generation	ND	ND	ND	ND	Diabetes
MESA; 2000–2018; Baltimore, MD, Chicago, IL, Forsyth County, NC, Los Angeles, CA, Northern Manhattan and Southern Bronx, NY, St. Paul, MN (11, 15, 96)	804 Asian, predominantly of Chinese descent^b^ (6,814 total)	Educational attainment Employment status and occupation Family income Home ownership	Nativity Language spoken at home Ethnic foods in diet	Discrimination	Usual source of care Health insurance	Perceived aspects of the neighborhood environment	2,154	Coronary heart disease and stroke
MEC; 1993–2022; Los Angeles, CA, state of Hawaii (65, 66, 122)^c^	70,892 predominandy Japanese and Native Hawaiian but also included Filipino, Samoan, and Korean (215,251 total)	Educational attainment Employment status and occupation	Nativity Years lived in the United States Ethnic foods in diet	ND	Geographic access to care	(Measures derived from secondary geospatial data) Structural racism (e.g., redlining) Neighborhood social and built environments Environmental factors	523	Cancer and other chronic diseases
MASALA; 2010–current; San Francisco, CA, Chicago, IL (54, 57)	1,164 Indian, Pakistani, Bangladeshi, Nepali, Sri Lankan	Educational attainment Employment status and occupation Family income Home ownership	Social or cultural gatherings Traditional cultural beliefs scale Spirituality and religiosity Nativity Years lived in the United States English language proficiency Language(s) spoken at home Types of food eaten at home/restaurant Frequency of shopping at South Asian markets Proportion of South Asian friends Frequency of social/cultural gatherings Frequency of fasting Traditional cultural beliefs scale Language of TV, radio, and print media Acculturation strategies	Everyday Discrimination Scale	Health care Insurance	Feelings about neighborhood safety, environment American Community Survey–linked neighborhood characteristics by geocoding	116	Atherosclerotic cardiovascular disease

Abbreviations: AANHPI, Asian American, Native Hawaiian, and Pacific Islander; CIL, Children of Immigrants Longitudinal Study; FHS, Filipino Health Study; HHP, Honolulu Heart Program; JACD, Japanese-American Community Diabetes Study; MASALA, Mediators of Atherosclerosis in South Asians Living in America; MEC, Multiethnic Cohort; MESA, Multiethnic Study of Atherosclerosis; ND, no data; UCSD, University of California, San Diego.

a Data on number of publications were either taken from cohort study pages or identified through PubMed through keyword searches and reflect all papers published from the cohort, not just those focusing specifically on AANHPI communities.

b While MESA describes Asian participants as Chinese Americans, this sample also consists of people who were born in many different Asian countries.

c Neighborhood factors reported for MEC included published linkages to publicly available sources of data and cohort supplements.

**Table 2:** Selected AANHPI cohort findings related to the effects of structural and social determinants on the health of AANHPI communities

Name of cohort	How has each study contributed to our understanding of how structural and social determinants of health impact the lives of AANHPI communities?					
	Socioeconomic status	Acculturation	Cultural/social constructs specific to AANHPI populations	Self-reported social stressors	Health care	Neighborhood environment
CIL (102, 103)	For children of immigrants (both Latin and Asian Americans), social capital minimally impacted economic adaptation (e.g., employment, use of public assistance), casting doubt on the theory of social capital as a determinant for this population (104).	Refugee families were less likely to aceulturate than were immigrants, which partially mediated the relationship between migration type and children’s beliefs about Asian family values (130). For Southeast Asian families, parental involvement in children’s home education, school, and social life fully explained the association between parental acculturation and intergenerational relationships (131).	Asian American children of immigrants have differences in beliefs about family values, with males hating more beliefs about the importance of taking jobs close to family and females hating more beliefs about asking relatives for help (121).	Southeast Asian American males were more likely to report perceptions and experiences of feeling unsafe in school than were females, as well as US-born (versus immigrant) youth (79, 124).	ND	Coethnic communities are an important resource for monolingual children, but less for bilingual children, because these spaces make coethnic information exchange accessible (75).
HHP (52, 71, 95, 106)	For Japanese men, occupational exposures to pesticides, metals, or solvents during midlife was associated with movement abnormalities, Parkinson disease (101), and increased mortality (16, 17).	Depression symptoms are significantly lower among elderly Japanese American men who are the most culturally Japanese (versus those who were more Westernized) (43). The use of spoken or written Japanese was not protective against cognitive decline (22). A longer amount of life spent in Japan and exposure to Japanese traditional culture reduced the risk of coronary heart disease later in life (129). Childhood exposure to Japan was negatively associated with cognitive test performance in late life; however, this association was not found in individuals who preferred being tested in Japanese, emphasizing the importance of using the subject’s native language for assessing cognitive function in older individuals (128).	Traditional beliefs about family obligation and care for elderly people at home offers a potential explanation for the greater rates of hospital death among Japanese Americans (9).	ND	ND	ND
UCSD FHS (5, 7)	Filipino women with low educational attainment and sustained social disadvantage had higher risk of type 2 diabetes (4).	ND	ND	ND	ND	ND
Seattle JACD	Early-life socioeconomic indicators (i.e., parental education, parental occupation) were associated with higher risk of type 2 diabetes (76).	ND	ND	ND	ND	ND
MESA (11, 15, 96)	A variety of socioeconomic factors, including education, occupational status, and income, were associated with performance on cognitive scores (31). Low socioeconomic position was found to be associated with biological markers of inflammation (105) and probability of a poor health outcome (e.g., cardiovascular disease, cognitive impairment) (114). Low socioeconomic status was associated with increased DNA methylation, which is related to stress and inflammation (92).	Increased assimilation into US culture and lower socioeconomic status were linked to a higher occurrence of carotid plaque and greater intima-media thickness but not to albuminuria, underscoring the importance of promoting healthy behaviors among recent immigrants (78). Among immigrants, greater time in the United States was associated with worse cardiovascular health (30, 97). In Chinese individuals, acculturation (greater time in the United States) was potentially protective, as indicated by reduced consumption of arachidonic acid (29). Among new immigrants, higher acculturation in the United States was associated with more myopia and less hyperopia (99).	ND	Experiences of discrimination were found to be associated with greater prevalence of smoking and alcohol consumption (12). Experiences of discrimination were associated with all-cause and cardiovascular mortality (73). Everyday discrimination was not found to be associated with incident diabetes (126). Associations between discrimination and inflammation were found to vary by gender and biological marker (62).	Having insurance, regardless of type, was linked to lower fasting glucose levels among individuals with diabetes in a diverse cohort, while variations in fasting glucose and insulin resistance were observed across different insurance types (38). Chinese participants in the fifth MESA examination were six times more likely to have no health insurance coverage compared with White participants (31).	Increased concentrations of air pollution were found to be associated with progression in coronary calcification, acceleration of atherosclerosis, decreased endothelial function, and subclinical interstitial lung disease (60, 70, 108). Residing in neighborhoods with better physical environments was associated with lower body mass index and could be a potential point of intervention for reducing obesity (86). Individual- and neighborhood-level measures of socioeconomic status were found to be associated with the concentration of air pollution (42). Residing in an immigrant community does not uniformly provide advantages and can be linked to diverse associations with various health behaviors (98). The quality of local food environments was associated with a higher probability of consuming organic foods (23).
MEC (65, 66, 122)	Socioeconomic position in childhood was associated with earlier pubertal onset (45). Native Hawaiians in Hawaii with low neighborhood socioeconomic status and education had higher all-cause mortality compared with Japanese Americans in Hawaii with high neighborhood socioeconomic status and education (109).	ND	ND	ND	ND	Lung cancer risk was positively associated with traffic-related air pollutants (18). Neighborhood socioeconomic status and obesogenic environment factors were found to be associated with prostate cancer risk (27). The risk of breast cancer is influenced by local residential segregation, which has varying effects on different racial/ethnic groups (64).
MASALA (54, 57)	In South Asian compared with White participants, sex, education, and insurance significantly contributed to the net difference in mean cardiovascular health score (110a).	Three distinct profiles of acculturation were observed among South Asians: separation (preferring South Asian culture), assimilation (preferring US culture), and integration (having similar preferences for both US and South Asian culture) (90). South Asian women with assimilation or integration acculturation strategies had better cardiometabolic profiles compared with those with separation (3). South Asian adults who had a separation acculturation strategy had more symptoms of depression compared with those who integrated (91). South Asian adults who had stronger self-rated American identities had worse cardiovascular health factors (118).	Among South Asians, the strength of traditional beliefs was associated with atherosclerosis, with those in the moderate traditional belief group hatting lower carotid intima-media thickness than those with higher or lower strength of beliefs (55). Those with Hinduism, Sikhism, and Muslim/Islam religious affiliation had higher odds of being overweight or obese compared with individuals without a religious affiliation (10). Muslims had higher levels of cholesterol compared with individuals with no religious affiliation (46). Religious struggles among South Asian Americans were found to moderate the relationship between specific protein concentrations and odds of cardiovascular disease (93). Both high- and low-self-rated religiousness and spirituality were positively associated with self-rated health (117).	Larger social networks were positively associated with ideal cardiovascular health (111). Experiences of discrimination were unrelated to fruit and vegetable intake but were associated with higher consumption of sweets (88).	ND	South Asian American men walked more minutes per week in neighborhoods that were more walkable (61). Neighborhood social cohesion was not associated with body mass index among South Asians (37) but was associated with reduced odds of hypertension (72).

Abbreviations: AANHPI, Asian American, Native Hawaiian, and Pacific Islander; CIL, Children of Immigrants Longitudinal Study; FHS, Filipino Health Study; HHP, Honolulu Heart Program; JACD, Japanese-American Community Diabetes Study; MASALA, Mediators of Atherosclerosis in South Asians Living in America; MEC, Multiethnic Cohort; MESA, Multiethnic Study of Atherosclerosis; ND, no data; UCSD, University of California, San Diego.

All papers referenced either focused on AANHPI populations or included this population in their analytic sample.
